# Correlations between baseline ^18^F-FDG PET tumour parameters and circulating DNA in diffuse large B cell lymphoma and Hodgkin lymphoma

**DOI:** 10.1186/s13550-020-00717-y

**Published:** 2020-10-07

**Authors:** Pierre Decazes, Vincent Camus, Elodie Bohers, Pierre-Julien Viailly, Hervé Tilly, Philippe Ruminy, Mathieu Viennot, Sébastien Hapdey, Isabelle Gardin, Stéphanie Becker, Pierre Vera, Fabrice Jardin

**Affiliations:** 1 Department of Nuclear Medicine, Henri Becquerel Cancer Centre, Rouen, France; 2 QuantIF-LITIS-EA4108, University of Rouen, Rouen, France; 3 Department of Haematology, Henri Becquerel Cancer Centre, Rouen, France; 4 INSERM U1245, Henri Becquerel Cancer Centre and Rouen University, Rouen, France

**Keywords:** Positron emission tomography, B cell malignancies, Diffuse large B cell lymphoma, Hodgkin lymphoma, Circulating tumour DNA, Circulating free DNA

## Abstract

**Background:**

^18^F-FDG PET/CT is a standard for many B cell malignancies, while blood DNA measurements are emerging tools. Our objective was to evaluate the correlations between baseline PET parameters and circulating DNA in diffuse large B cell lymphoma (DLBCL) and classical Hodgkin lymphoma (cHL).

**Methods:**

Twenty-seven DLBCL and forty-eight cHL were prospectively included. Twelve PET parameters were analysed. Spearman’s correlations were used to compare PET parameters each other and to circulating cell-free DNA ([cfDNA]) and circulating tumour DNA ([ctDNA]). *p *values were controlled by Benjamini–Hochberg correction.

**Results:**

Among the PET parameters, three different clusters for tumour burden, fragmentation/massiveness and dispersion parameters were observed. Some PET parameters were significantly correlated with blood DNA parameters, including the total metabolic tumour surface (TMTS) describing the tumour–host interface (e.g. *ρ* = 0.81 *p* < 0.001 for [ctDNA] of DLBLC), the tumour median distance between the periphery and the centroid (medPCD) describing the tumour’s massiveness (e.g. *ρ* = 0.81 *p* < 0.001 for [ctDNA] of DLBLC) and the volume of the bounding box including tumours (TumBB) describing the disease’s dispersion (e.g. *ρ* = 0.83 *p* < 0.001 for [ctDNA] of DLBLC).

**Conclusions:**

Some PET parameters describing tumour burden, fragmentation/massiveness and dispersion are significantly correlated with circulating DNA parameters of DLBCL and cHL patients. These results could help to understand the pathophysiology of B cell malignancies.

## Background

B cell malignancies include non-Hodgkin's lymphomas (NHL), with diffuse large B cell lymphoma (DLBCL) which accounts for approximately one third to one half of NHL in adults [1] and classical Hodgkin lymphoma (cHL). NHL and cHL share the same cell of origin and are among the most frequent cancers with, for NHL, an incidence in the USA of 19.6 per 100,000 [2] and, for cHL, an incidence of 2.7 per 100,000 per year [3]. These two diseases are separated as two distinct categories in World Health Organization (WHO) classification due to major differences that were well described in morphology, phenotype, epigenetic, natural history, treatment strategies and clinical manifestations.

In both DLCBL and cHL, ^18^F-fluorodeoxyglucose (FDG) positron emission tomography coupled with computed tomography (PET/CT) is a standard at baseline to describe the extension of the disease, but also during the follow-up to evaluate the therapeutic response [4]. Among the different parameters which can be extracted on the PET/CT, the tumour burden explored by total metabolic tumour volume (TMTV) is a well-known prognostic parameter for both DLBCL and cHL [5]. With the development of the radiomics [6], more complex quantitative parameters than TMTV can be extracted from imaging and analysed, some of them describing meaningful tumour phenotype. However, if thousands of different radiomical features, including “textural” features, can be extracted and analysed from medical images [6, 7], most of them can be applied only on unique tumours, while lymphomas are, most of the time, multisite tumours and it is possible that textural features are therefore less effective in lymphomas. Therefore, if entropy, a parameter among the textural features exploring the relationships between the tumour pixels, seems to be a prognostic factor for mantle cell lymphoma [8, 9], it is usually difficult to extract and analyse “textural” parameters when multiple tumours are considered because they are mathematically designed for unique tumour [10]. For multisite tumours, like lymphomas, parameters describing the tumour burden, tumour fragmentation/massiveness, tumour dispersion and tumour activity seem particularly relevant. Therefore, new parameters have proven to have a prognostic value [11, 12], notably the tumour volume surface ratio (TVSR) describing the tumour fragmentation and which is an independent prognostic factor in DLBCL and has an additional prognostic value when combined with TMTV, international prognostic index (IPI) score and type of chemotherapy used [12]. However, in order to optimally describe the tumour phenotype in imaging, many new parameters have to be defined and evaluated.

In parallel with FDG PET/CT, biology has also made great progress in describing lymphomas, notably the concept of liquid biopsy. One major tool of liquid biopsy is the analysis of circulating tumour DNA (ctDNA), which is the tumour fraction of plasma cell-free circulating DNA (cfDNA), measured by the variant allele frequencies (VAF) of somatic tumour mutations. In healthy subjects, cfDNA is detectable as low level due to the normal apoptosis of nucleated cells, in particular hematopoietic cells. Therefore, the detection of ctDNA requires highly sensitive technologies, such as next-generation sequencing (NGS) approaches or digital PCR (dPCR), capable of detecting low-frequency somatic variants. In cancerous patients, plasma ctDNA is released by apoptotic tumour cells, by necrotic tumour cells or is actively secreted by tumour cells [13] and ctDNA level can have a prognostic value at baseline, notably for DLBCL [14]. To quantify the ctDNA level ([ctDNA]), we have recently designed and validated two sets of genes for DLBCL (panel of 34 genes frequently mutated in DLBCL patients, so-called lymphopanel) [15] and for cHL (set of 9 genes) [16]. In these previous studies, [ctDNA] was correlated with factors of poor prognosis, notably international pronostic index (IPI) for DLBCL and stage 3–4, anaemia and symptoms B for cHL. Moreover high TMTV and high [ctDNA] level were found to be significantly correlated at the time of diagnosis for both DLBCL and cHL [15, 16]. However, these correlations were moderate, suggesting that other factors may influence these two approaches of tumour burden quantification. Thus, the links between the tumour phenotype (tumour burden, tumour fragmentation/massiveness, tumour dispersion and tumour activity), which can be determined on baseline PET/CT, and circulating DNA have yet to be explored, notably to explain the pathophysiology of ctDNA release.

The main objective of this study was to evaluate the correlations between baseline PET parameters and circulating DNA levels ([cfDNA] and [ctDNA]) in two B cell malignancies: DLBCL and cHL.

## Results

### Patients

Thirty patients with diffuse large B cell lymphoma (DLBCL) and 50 patients with classical Hodgkin lymphoma (cHL) were enrolled prospectively. Description of the population with clinical characteristics and outcomes is available in two previous studies [15, 16]. All patients had performed a PET/CT on one of 3 different machines (description summarized in Additional file 1: Table 1). As one machine (PET/CT n°3) represented only 6,25% of the examinations performed (*n* = 3 for DLBCL and *n* = 2 for cHL), the patients concerned were excluded to avoid a bias linked to the PET/CT used. Description of the population studies, values of PET parameters and blood cell-free DNA measurements is available in Table 1. For the analysis concerning ctDNA, 3 patients for DLBCL and 15 patients for cHL were excluded as no mutation corresponding to lymphoma was found in ctDNA (false negatives of the technique at baseline).

**Table 1:** Patients characteristics and values of PET and blood DNA parameters

	DLBCL (*n* = 27) *n* (%)	cHL (*n* = 48) *n* (%)
Median age [range] (in years)	69 [20–93]	33 [20–86]
Male	15 (55.5%)	22 (45.8%)
Stage III/IV (vs. I/II)	20 (74%)	20 (41.7%)
LDH > ULN	10 (37%)	8 (17%, NA = 1)
Spleen involvement	2 (7%)	3 (6%)
Median time interval between blood sample and PET [maximal] (in days)	9 [61]	8 [65]

	PET parameters mean (± SD) [min–max]	
(1) SUVmax	22.4 (± 8.3) [4.3–35.5]	15.4 (± 6.2) [3.6–33.8]
(2) SUVmean	10.3 (± 3.6) [2.9–17.3]	5.7 (± 1.8) [2.2–11.3]
(3) TMTV (cm^3^)	761 (± 898) [0.2–2927]	280 (± 291) [2–1256]
(4) TLG	8596 (± 10,068) [0.7–30,773]	1537(± 1444) [5–6520]
(5) TMTS (cm^2^)	910 (± 1017) [2–3309]	570 (± 570) [12–3218]
(6) TVSR (mm)	7.0 (± 3.4) [1.2–15.2]	4.5 (± 1.6) [1.9–9.6]
(7) TumBB (cm^3^)	13,997 (± 18,021) [0–60,982]	12,961 (± 22,462) [6–107,039]
(8) Dmax (mm)	404 (± 294) [10–1026]	376 (± 313) [26–1175]
(9) nROI	9.2 (± 12.6) [1–65]	12 (± 10.8) [1–49]
(10) itErosion	2.2 (0.8) [1–4.46]	1.6 (± 0.4) [1.0–2.8]
(11) medPCD (mm)	49.8 (± 29.7) [3.9–117.5]	29.6 (± 15.1) [7.7–80.1]
(12) medEdgeD (mm)	44.6 (± 20.2) [3.3–79.4]	29.2 (± 10.2) [11.3–57.6]

	Blood cell-free DNA parameters mean (± SD) [min–max]	
[cfDNA] (hGE/mL)	22,665 (± 28,414)[4206–124,425]	13,771 (± 18,975)[2023–88,046]
[ctDNA] (hGE/mL)	4961 (± 9948) [2–39,151] (3 NA, no mutation found)	335 (± 623)[10–2684] (15 NA, no mutation found)

*cfDNA* circulating free DNA, *cHL* classical Hodgkin's lymphoma, *ctDNA* circulating tumour DNA, *DLBCL* diffuse large B cell lymphoma, *LDH* lactate dehydrogenase, *NA* not available, *PET* positron emission tomography

### Distribution of correlations between PET parameters with each other

Comparison of PET parameters distribution according to the machine use is summarized in Additional file 1: Table 1. No statistically significant difference (Wilcoxon–Mann–Whitney test *p *values > 0.05) was observed in the distribution of all PET parameters for DLBCL between PET/CT n°1 (*n* = 14) and PET/CT n°2 (*n* = 13). For cHL, only four parameters (TumBB, Dmax, itErosion, medPCD) among the twelve studied had a statistically significant difference (Wilcoxon–Mann–Whitney test *p *values < 0.05) in the distribution between PET/CT n°1 (*n* = 14) and PET/CT n°2 (*n* = 34). For these four parameters, a post-reconstruction harmonization by ComBat [17, 18] was performed to avoid a bias linked to PET/CT used (see Additional file 2: Fig. 1), with no statistically significant difference found between the distributions after harmonization (Wilcoxon–Mann–Whitney test *p *values > 0.05) and the results obtained used for the subsequent analysis.

**Figure 1:**
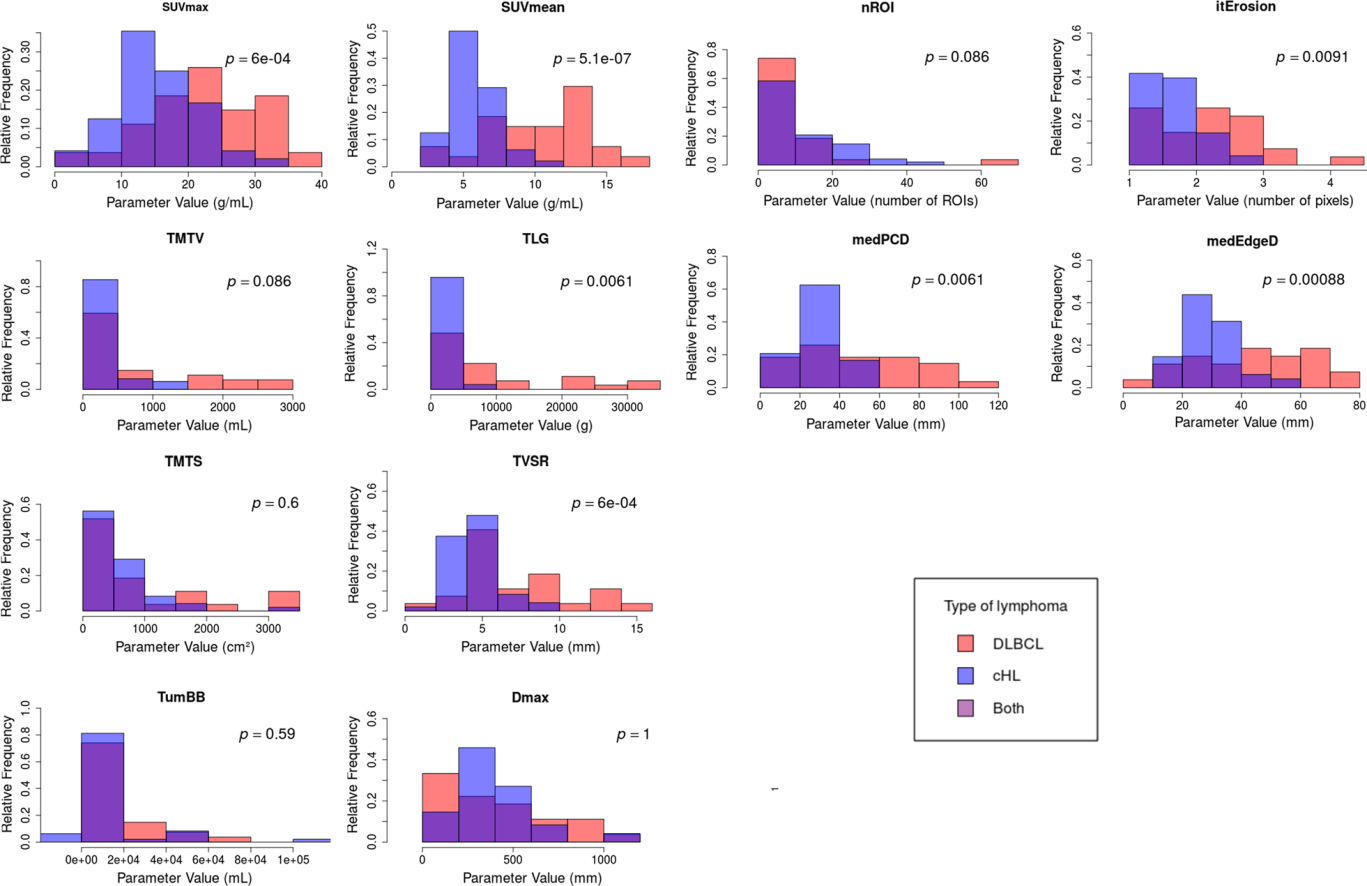
Plots representing distribution histograms of PET parameters fused for DLBCL and cHL with Wilcoxon–Mann–Whitney test *p *value

Histograms of the distributions of each PET parameter, fused for DLBCL and cHL, are presented in Fig. 1. TMTV, TMTS, TumBB, Dmax and nROI showed non-different distributions for both DLCBL and cHL (Wilcoxon–Mann–Whitney test *p *values > 0.05), while the distributions were significantly different for the other parameters (Wilcoxon–Mann–Whitney test *p *values < 0.05).

**Figure 2:**
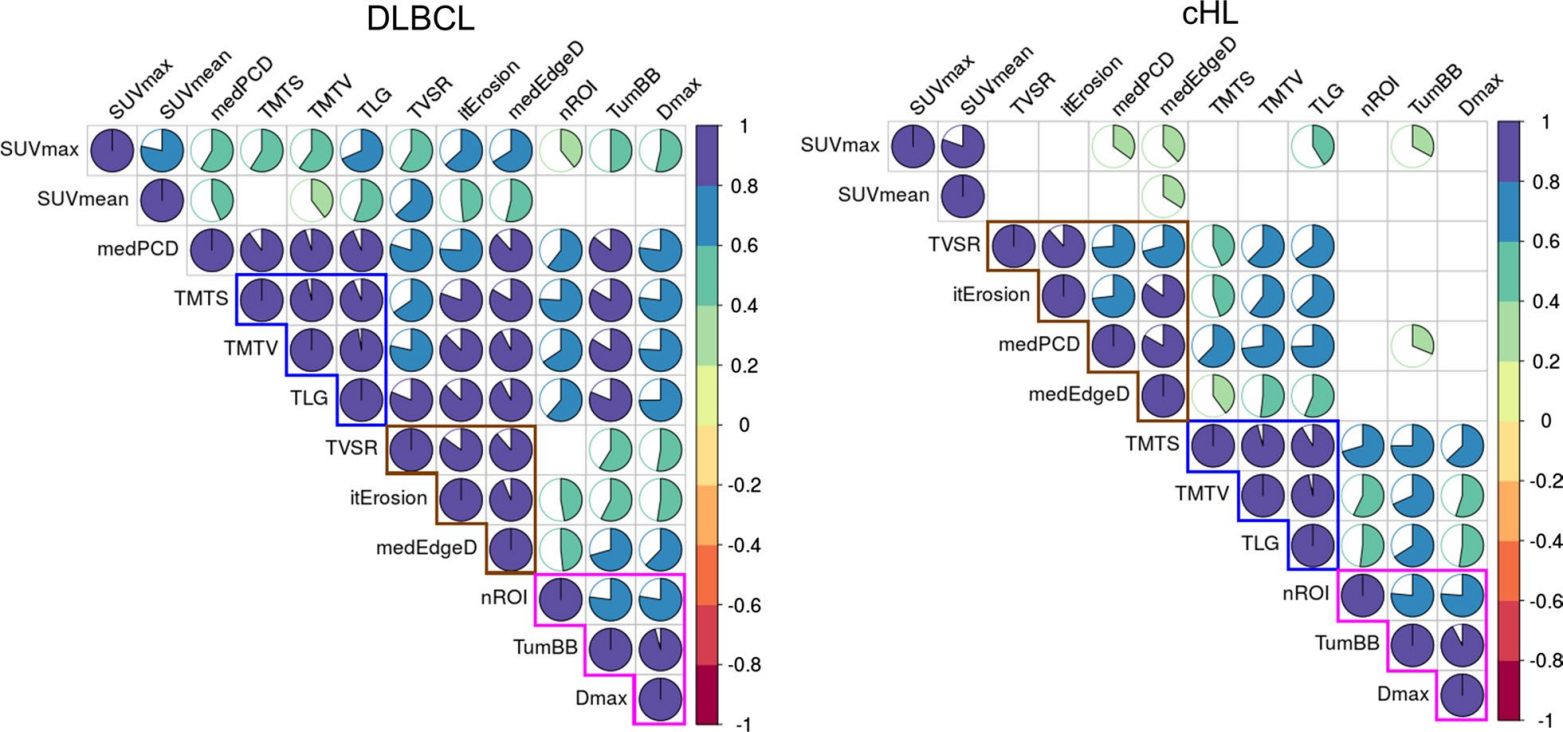
Plot representing clusters of Spearman’s correlations (with *p*<0.05 controlled by Benjamini–Hochberg correction) between PET parameters for DLBCL and cHL. The blue, brown and rose lines show the three similar clusters found in both DLBCL and cHL

Plots of high Spearman’s correlations (*p* < 0.05 controlled by Benjamini–Hochberg–BH correction [19]) between PET parameters for DLBCL and cHL are presented in Fig. 2. Three similar clusters were found in both DLBCL and cHL combining highly correlated parameters: the first one concerning the tumour burden with TMTV, TMTS and TLG, the second describing the tumour dispersion with TumBB and Dmax (and also with nROI with a lower correlation) and the third concerning the massiveness with medEdgeD and itErosion (but also TVSR and medPCD, which is, in the case of DLCBL, also associated with the tumour burden). MedEdgeD, itErosion, TumBB were also well correlated with TMTV and TLG for DLBCL. All statistically significant correlation values are visible in Additional file 3: Fig. 2.

### Correlations between PET parameters and circulating DNA ([cfDNA] and [ctDNA])

Table 2 represents the Spearman’s correlations between the twelve PET parameters and the two blood DNA parameters ([cfDNA], [ctDNA]) for DLBCL and cHL, with the 3 first higher values parameters with an asterisk for each category (*p *values controlled by BH correction).

**Table 2:** Spearman’s correlations between twelve PET parameters (SUVmax, SUVmean, TMTV, TLG, TMTS, TVSR, TumBB, Dmax, nROI, itErosion, medPCD, medEdgeD) and two blood DNA parameters ([cfDNA], [ctDNA]) for DLBCL and cHL and with the 3 more important and significant values with an asterisk for each categories

	[cfDNA]				[ctDNA]			
	DLCBL		cHL		DLBCL		cHL	
	*ρ*	*p *value	*ρ*	*p *value	*ρ*	*p *value	*ρ*	*P *value
(1) SUVmax	0.44	0.02	0.10	0.77	0.41	0.05	0.30	0.14
(2) SUVmean	0.18	0.38	-0.06	0.80	0.19	0.37	0.08	0.74
(3) TMTV	0.76*	< 0.001	0.33	0.14	0.79	< 0.001	0.55*	0.005
(4) TLG	0.74*	< 0.001	0.31	0.14	0.75	< 0.001	0.58*	0.005
(5) TMTS	0.78*	< 0.001	0.36	0.14	0.81*	< 0.001	0.52	0.005
(6) TVSR	0.50	0.009	0.08	0.77	0.56	0.006	0.26	0.20
(7) TumBB	0.69	0.001	0.27	0.17	0.83*	< 0.001	0.54*	0.005
(8) Dmax	0.67	< 0.001	0.23	0.21	0.77	< 0.001	0.50	0.007
(9) nROI	0.66	< 0.001	0.26	0.17	0.75	< 0.001	0.41	0.03
(10) itErosion	0.59	0.001	0.05	0.8	0.59	0.003	0.17	0.42
(11) medPCD	0.70	< 0.001	0.25	0.81	0.81*	< 0.001	0.41	0.03
(12) medEdgeD	0.62	< 0.001	0.04	0.17	0.65	< 0.001	0.02	0.91

*p *values controlled by Benjamini–Hochberg correction

Concerning cfDNA and for DLBCL, three PET burden parameters (TMTS, TMTV and TLG) had the highest correlations with [cfDNA] (*ρ* = 0.78, *ρ* = 0.76 and *ρ* = 0.74, respectively). Parameters of dispersion, like TumBB, and of massiveness, like medPCD, were also well correlated (*ρ* = 0.69 and *ρ* = 0.70, respectively). For cHL, no statistically significant correlation was found between PET parameters and [cfDNA].

Concerning ctDNA, dispersion parameter TumBB had correlation values with [ctDNA] among the highest for both DLBCL (*ρ* = 0.83) and cHL (*ρ* = 0.54). For DLBCL, medPCD, describing massiveness and TMTS were also highly correlated with [ctDNA] (*ρ* = 0.81 for both). For cHL, two burden parameters, TLG and TMTV, presented the highest correlation values with [ctDNA] (*ρ* = 0.58 and *ρ* = 0.55, respectively).

In an univariate linear regression analysis (see Additional file 4: Table 2), most of the parameters were significantly associated with [cfDNA] of DLBCL and none to [cfDNA] of cHL. Concerning [ctDNA], burden parameters (TMTV, TMTS and TLG) were significantly associated with it in both DLBCL and cHL. Dispersion parameters (TumBB and Dmax) were significantly associated with [ctDNA] in cHL and massiveness parameters (itErosion, medPCD, medEdgeD) with [ctDNA] in DLBCL.

In the multivariate stepwise regression analysis used to determine [cfDNA] and [ctDNA] from PET parameters (see Table 3), statistically significant formulas were found for both DLBCL and cHL even if adjusted *R*^2^ was higher for DLBCL (0.53 and 0.44 for [cfDNA] and [ctDNA], respectively) than for cHL (0.28 and 0.40 for [cfDNA] and [ctDNA], respectively). Interestingly, TMTS and medEdgeD were associated with determined [ctDNA] in both DLBCL and cHL, while TMTV was absent of the formulas used to predict [ctDNA].

**Table 3:** Multivariate stepwise regression between twelve PET parameters (SUVmax, SUVmean, TMTV, TLG, TMTS, TVSR, TumBB, Dmax, nROI, itErosion, medPCD, medEdgeD) and two blood DNA parameters ([cfDNA], [ctDNA]) with adjusted *R*-squared (*R*^2^) and *p* values

[cfDNA]		[ctDNA]	
DLBCL	cHL	DLBCL	cHL
(Intercept) − 7.78e03 + TMTV*20.1 + itErosion*2.25e04 + medEdgeD* − 7.33e02	(Intercept) 1.89e04 + TMTV* − 6.14e01 + TMTS*5.25e01 + TVSR*1.05e04 + TumBB* − 5.28e−01 + Dmax*6.04e01 + nROI* − 1.05e03 + itErosion* − 4.18e04	(Intercept) − 7.72e03 + itErosion*9.95e03 + TMTS*5.06 + medEdgeD* − 2.86e02	(Intercept) 3.62e02 + TMTS*7.82e − 01 + TVSR*2.09e02 + TumBB* − 2.55e−02 + Dmax*2.72e00 + nROI* − 4.41e01 + medPCD*4.31e01 + medEdgeD* − 9.28e01
[*R*^2^ = 0.53] (*p* < 0.001)	[*R*^2^ = 0.28] (*p* = 0.006)	[*R*^2^ = 0.44] (*p* = 0.002)	[*R*^2^ = 0.40] (*p* = 0.005)

## Discussion

In this study analysis patients prospectively included, we explored the correlations between PET parameters and circulating DNA ([cfDNA] and [ctDNA]) in two B cell malignancies, DLBCL and cHL. We have seen that TMTV, a tumour burden parameter, but also some other PET parameters, describing tumour burden, fragmentation/massiveness and dispersion, were significantly correlated with cfDNA and ctDNA levels. These parameters could be associated to predict cfDNA and ctDNA release for both DLBCL and cHL as shown by a multivariate analysis.

The PET parameters studied explored different phenotypes of the tumours: the tumour burden (with TMTV, TMTS but also TLG as it is a parameter highly correlated with the two previous), the tumour activity (SUVmax, SUVmean and, partly, TLG), the tumour dispersion (Dmax, TumBB) and the tumour massiveness/fragmentation (TVSR, medPCD, medEdgeD, nRoi, itErosion). These parameters consider the disease as a whole and can characterize all tumour locations. This type of parameters is well suited to some cancers, notably lymphomas and probably metastatic solid cancers, as shown by the predictive value of known parameters like TMTV [5], TVSR [12] or Dmax [20].

Concerning the burden parameters, TMTV was found to be significantly correlated with [cfDNA] and [ctDNA] levels in both DLBLC and cHL, as already found in previous studies [15, 16]. We have also found that TMTS was frequently among the more highly correlated parameters with cfDNA and ctDNA levels (among the three first parameters in 2 cases of 4). It was notably highly correlated with [ctDNA] (*ρ* = 0.81 *p* < 0.001) for DLBCL. One possible pathophysiological explanation is that TMTS represents the tumour surface where the progression or the regression of the tumours [21] occurs and, therefore, the “battlefront” between the tumour and the host where cytolysis takes place.

For tumour massiveness/fragmentation parameters, medPCD, corresponding to the median distance between the centroid of the tumours and the periphery, was also among the more highly correlated parameter with [cfDNA] and [ctDNA] for DLBCL (*ρ* = 0.70 and *ρ* = 0.81, respectively). One possible hypothesis for this link between massiveness and circulating blood DNA is the fact that massive tumour can be necrotic at their centre and therefore release cell-free DNA in the bloodstream.

Among the dispersion parameters, TumBB, representing the dispersion volume of the tumours, was among the more highly correlated parameter with [ctDNA] for both DLBCL and cHL (*ρ* = 0.83 and *ρ* = 0.54, respectively). One explanation is the fact that TumBB is linked to the extend and the stage of the disease, like Ann Arbor staging.

In this study, we explored PET parameters dedicated to multisite tumours to determine the tumour burden, activity, dispersion and massiveness/fragmentation. These parameters belong to the “shape” and “intensity” parameter types [22]. Some other parameters, including “textural” parameters used to determine the heterogeneity of the tumours [9], could be interesting with possible links between DNA parameters (in bloodstream or in tumour biopsy) and PET parameters. However, most of the “textural” parameters are mathematically designed to characterize unique tumour [10, 23] and have therefore to be applied on the largest lymphoma lesion which could be representative of the disease, notably to predict survival [24].

It has to be noticed that, contrary to the “textural” ones, the parameters based on “shape” and “intensity”, such as those explored in our study, are robust and less sensitive to PET/CT machine and reconstruction used [17, 22, 25]. This is concordant with the Wilcoxon–Mann–Whitney tests performed in our study comparing the PET parameters distribution acquired by the two different machines: in both diseases, no statistically significant difference was observed in the distribution of 83% of the parameters studied (100% if considering DLBCL alone). For the remaining 17% (only 4 parameters of cHL), we applied a data harmonization by the ComBat method [17, 18] to avoid a potential bias linked to the difference of the machines used. However, the robustness, notably inter-observer one, and reproducibility of the global parameters, based on “shape” and “intensity”, compared to other parameters, such as “textural” parameters, have to be confirmed by other studies. Moreover, the prognostic value of these parameter has to be explored on a larger population with a sufficient follow-up. To allow this, and in accordance with the “image biomarker standardisation initiative” (IBSI) guidelines [23], we have described the PET parameters studied, as well as the acquisitions performed, in such a way that they can be reproduced by other teams.

There is a growing trend towards a PET/[ctDNA] combination to monitor minimal residual disease (MRD) in patients, hence the relevance of this work. In addition, PET parameters explored by Oncometer3D can be easily calculated, automatically and quickly from tumour contours. This type of new parameters will become more and more accessible as tumours are increasingly segmented automatically [26, 27] and could help to determine whether ctDNA should replace or be additional data to PET/CT.

## Conclusions

Some PET parameters describing tumour burden, fragmentation/massiveness and dispersion are significantly correlated with the blood DNA parameters of DLBCL and cHL. These results could help to understand the pathophysiology of these B cell malignancies. In addition, the combination of PET parameters and liquid biopsy could improve patient monitoring.

## Materials and methods

### Patients

Patients with DLBCL and cHL were enrolled prospectively in two non-interventional studies: LymphoSeq (NCT 02339805) and XPO1 (NCT 02815137). Clinical features and biological material at the time of diagnosis, including DNA from blood, were collected before any treatment. Patients were followed after rituximab-cyclophosphamide-doxorubicin-vincristine-prednisone (R-CHOP) or R-CHOP-like chemotherapies for DLBCL, or after ABVD or BEACOPP-escalated chemotherapies for cHL. An ^18^FDG-PET/CT was performed at the time of diagnosis and during the follow-up (mid-treatment and end of treatment, according to treatment strategies).

Patients provided written informed consent in accordance with the Declaration of Helsinki, and the Institutional Review Board of Henri Becquerel Cancer Centre approved the protocol (registration clinical.gov numbers: NCT02339805 and NCT02815137).

### Blood specimens

Blood samples were obtained by blood tests at diagnosis on EDTA tubes and were centrifuged for 10 min at 3000–3500 rpm within three hours of collection. Plasma was aliquoted into 1 mL in microtubes and stored at -80 °C until extraction.

Circulating cell-free DNA ([cfDNA]) was extracted from 3 mL of plasma aliquots with Amp Circulating Nucleic Acid® QI Kit (Qiagen, Hilden, Germany) according to the manufacturer's instructions and quantified using QuBit High Sensitivity dsDNA (ThermoFisher Scientific, Illkirch, France). After tumour sequencing adapted to the type of cancer [15, 16], the DNA was eluted in 60 to 80 μL of AVE buffer and then stored at − 80 °C. Quantification of the double-stranded DNA was performed by fluorometry on Qubit 2.0 (ThermoFisher Scientific Carlsbad, CA, USA), with Qubit® dsDNA kit HS Assay (ThermoFisher Scientific, Carlsbad, CA, USA). The circulating cell-free tumour DNA ([ctDNA]) concentrations were expressed in haploid genome equivalents per mL of plasma (hGE/mL) and calculated by multiplying the mean variant allelic frequency (VAF) for all mutations used for detection calling by the concentration of [cfDNA] (pg/mL of plasma) and dividing by 3.3, using the assumption that each haploid genomic equivalent weighs 3.3 pg, as previously described in the publication by Scherer et al. [28].

### Positron emission tomography (PET) analysis

All patients underwent FDG PET/CT before the onset of chemotherapy, performed after a 6-h fasting and when blood glucose level was less than 1.7 g/L. PET data were acquired on 3 different PET systems, approximately 60 min after injection of 3.5 to 4.5 Mbq/kg, from the mid-thigh toward the base of the skull, 3 to 4 min per bed position. CT scan was set up to 100 to 120 kV, with an intensity modulation system. Injected activity, acquisition time and CT parameters were depended of the PET system and patient’s habitus. No contrast agent was administered to the patients for CT. Three different PET/CT scanners were used without selection according to patients: a Biograph16 HiRes (Siemens®, Germany) (PET/CT n°1), a GE710 (General Electrics®, USA) (PET/CT n°2) and a Biograph40 mCT (Siemens®, Germany) (PET/CT n°3) with acquisition parameters described in Additional file 1: Table 1. PET system was normalized daily and the calibration coefficient validated if the day-to-day variation remained below 0.3%. The global quantification, from the dose calibrator to the imaging system, was measured internally on a quarterly basis and double checked by the EARL’s quality assurance program. SUV was normalized according to the weight of the patients. Regions of interest were segmented semi-automatically with PET VCAR (General Electrics®, USA) based on 41% SUVmax segmentation, by consensus of two nuclear medicine physicians (SB and PD) with a visual control and manual adaptation if necessary. The spleen was considered as involved if there was focal uptake or diffuse uptake higher than 150% of the liver background. The bone marrow involvement was only included in the volume measurement if there was focal uptake. Contours (in RTSS format) were converted to binary mask (in mha format) by using the software plastimatch [29]. Two images mask was used for each patient: one binary mask with only contours, one 32-bits mask with SUV values hard-coded in the pixel data. PET parameters were extracted by using the in-house software Oncometer3D version 1.0.

### PET parameters

**Figure 3:**
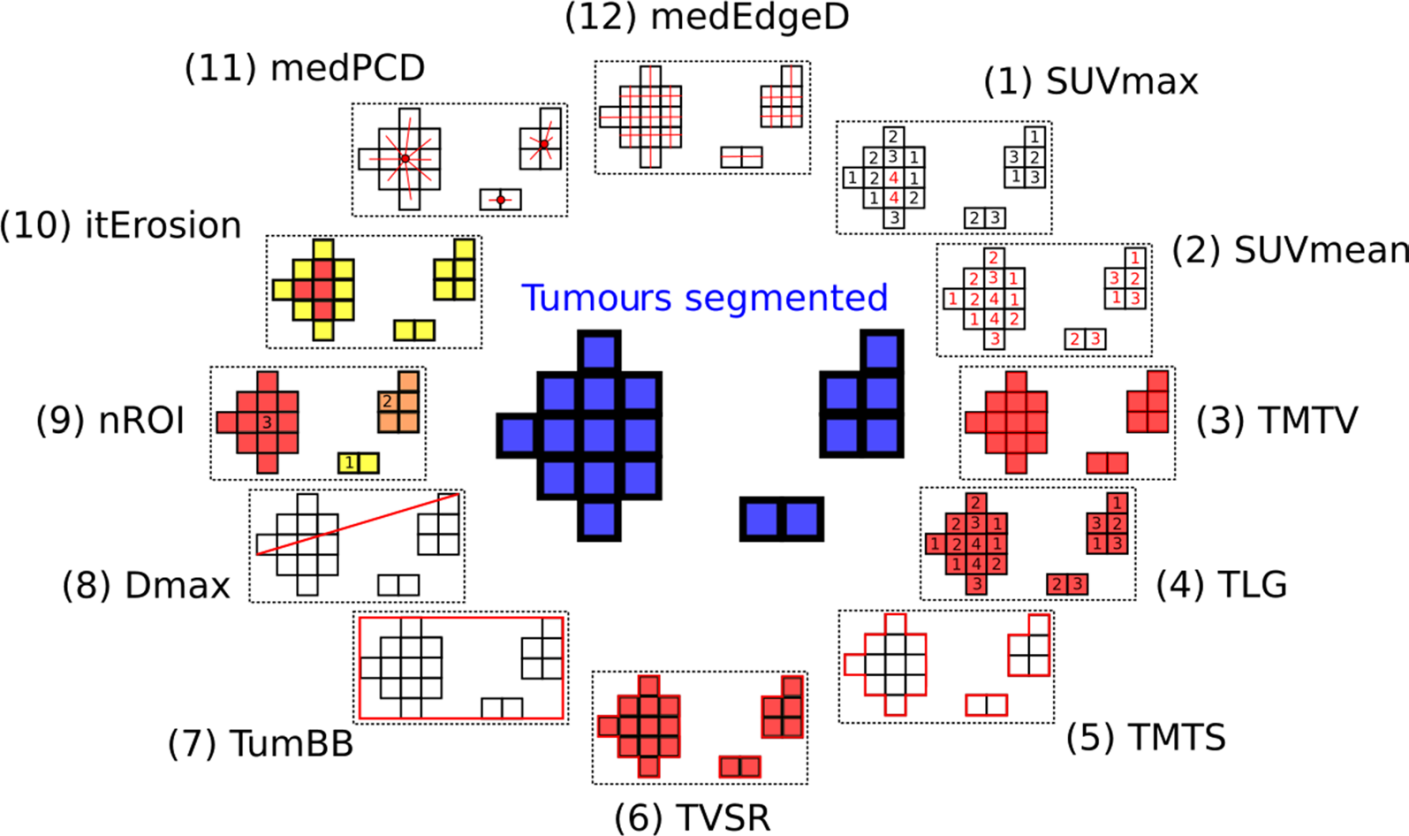
Representation of the twelve different PET parameters measured by the software Oncometer3D and analysing burden, activity, dispersion, fragmentation and massiveness of the lymphoma. For graphical purpose, a planar representation of these 3D parameters is shown

The twelve following parameters were determined on the baseline PET/CT by an in-house software called “Oncometer3D”, with a graphical representation of the parameters visible in Fig. 3: (1) SUVmax, the highest maximal standardized uptake value (SUV) measured in all tumours, (2) SUVmean, the mean value of SUV measured in all tumours, (3) total metabolic tumour volume (TMTV) obtained by summing the metabolic volumes of all the nodal and extra-nodal lesions, (4) total lesion glycolysis (TLG) calculated as the product of the MTV and the SUVmean (TLG = TMTV × SUVmean), (5) total metabolic tumour surface (TMTS) obtained by summing the metabolic surfaces of all tumours [12], (6) tumour volume surface ratio (TVSR) corresponding to the ratio of the TMTV and the TMTS [12], (7) volume of the bounding box including the tumours (TumBB) corresponding to the volume of tumour dispersion, (8) the maximal tumour distance (Dmax) corresponding to the distance between the two lesions that were the furthest apart [20], (9) the number of regions of interest (nROI) corresponding to the number of unique tumour on the whole examination, (10) iterative erosion (itErosion) corresponding to the number of erosions [30] required to remove tumours from the images, (11) the median distance between the centroid of the tumours and the periphery (medPCD), (12) the median edge distance (medEdgeD) corresponding to the median distance between the opposite edges of the tumours.

### Statistical analysis

In order to analyse a potential “machine effect” due to the use of several different PET/CT machines, the distributions of the different PET parameters according to the machines used and the type of disease were compared by Wilcoxon–Mann–Whitney tests. In case of differences in the distributions, a harmonization by ComBat method was carried out [17, 18].

To illustrate and compare distribution of PET parameters between DLBCL and cHL, fused histograms were produced with Wilcoxon–Mann–Whitney tests performed between the distributions of DLBCL and those of cHL.

To characterize the relationships between the different parameters, Spearman’s rank correlation coefficient of PET/CT parameters with each other and with DNA parameters ([cfDNA], [ctDNA]) was calculated separately for DLBC and cHL. For [ctDNA], null values were excluded from the analysis as they correspond to a false negative (mutation of the lymphoma not present in the panel and, therefore, no possibility to measure the ctDNA).

An univariate linear regression analysis was performed to explore linear correlations between PET and DNA parameters. To explore the interest of the combination of several factors, a multivariate stepwise regression analysis in both direction was used to determine [cfDNA] and [ctDNA] from the twelve parameters for both diseases.

Statistical significance was considered at *p* < 0.05 controlled by Benjamini–Hochberg correction [19]. All statistical analyses were performed using R software version 3.4.4 [31].

## Supplementary information

**Additional file 1:** **Table 1**. Acquisition parameters of the 3 different PET/CT used and Wilcoxon test p-values between distributions of PET parameters for DLCBL and cHL according to the acquisition on PET/CT n°1 (Biograph16) or PET/CT n°2 (GE710). *p *values controlled by Benjamini–Hochberg correction.

**Additional file 2:** **Figure 1.** Plot representing distribution of four PET parameters (TumBB, Dmax, itErosion, medPCD) acquired on PET/CT n°1 and n°2 before and after harmonization by ComBat with Wilcoxon–Mann–Whitney test p-value.

**Additional file 3:** **Figure 2**. Plot representing Spearman’s correlations (*p *< 0.05 controlled by Benjamini–Hochberg correction, else blank) between PET parameters for DLBCL and cHL.

**Additional file 4:** **Table 2.** Univariate linear regression between twelve PET parameters (SUVmax, SUVmean, TMTV, TLG, TMTS, TVSR, TumBB, Dmax, nROI, itErosion, medPCD, medEdgeD) and two blood DNA parameters ([cfDNA], [ctDNA]) for DLBCL and cHL with adjusted *R*-squared (*R*²). *p *values controlled by Benjamini–Hochberg correction.

## Data Availability

The datasets used and/or analysed during the current study are available from the corresponding author on reasonable request.

## Abbreviations

AUC: Area under the curve
BH: Benjamini–Hochberg
cfDNA: Circulating cell-free DNA
cHL: Classical Hodgkin lymphoma
ctDNA: Circulating tumour DNA
DLBCL: Diffuse large B cell lymphoma
dPCR: Digital PCR
itErosion: Iterative erosion
Dmax: Maximal tumour distance
medEdgeD: Median edge distance
MRD: Minimal residual disease
NGS: Next-generation sequencing
NHL: Non-Hodgkin's lymphomas
nROI: Number of regions of interest
PET: Positron emission tomography
ROC: Receiver operating characteristic
SUV: Standardized uptake value
TLG: Total lesion glycolysis
TMTS: Total metabolic tumour surface
TMTV: Total metabolic tumour volume
medPCD: Tumour median distance between the periphery and the centroid
TVSR: Tumour volume surface ratio
VAF: Variant allele frequencies
volBB: Volume of the bounding box including tumours
