# Supplementary material for: Correlations between baseline 18F-FDG PET tumour parameters and circulating DNA in diffuse large B cell lymphoma and Hodgkin lymphoma
Source: EJNMMI Res. 2020 Oct 7;10:120. doi: 10.1186/s13550-020-00717-y (PMC7541805; doi:10.1186/s13550-020-00717-y)
Supplement: Supplementary file 1 — Additional file 1: Table 1. Acquisition parameters of the 3 different PET/CT used and Wilcoxon test p-values between distributions of PET parameters for DLCBL and cHL according to the acquisition on PET/CT n°1 (Biograph16) or PET/CT n°2 (GE710). p values controlled by Benjamini–Hochberg correction. [file 13550_2020_717_MOESM1_ESM.doc]

**Supplemental table 1**. Acquisition parameters of the 3 different PET/CT used and Wilcoxon test p-values between distributions of PET parameters for DLCBL and cHL according to the acquisition on PET/CT n°1 (Biograph16) or PET/CT n°2 (GE710). P-values controlled by Benjamini-Hochberg correction.

|  | Acquisition parameters  of the 3 different PET/CT | | | Wilcoxon test p-values  between distributions of PET parameters | | |
| --- | --- | --- | --- | --- | --- | --- |
|  | PET/CT n°1 | PET/CT n°2 | PET/CT n°3 |  | DLBCL | cHL |
| Name | Biograph16 HiRes | GE710 | Biograph40 mCT | Number of examinations | PET/CT n°1, n=14  PET/CT n°2, n=13 | PET/CT n°1, n=14  PET/CT n°2, n=34 |
| Manufacturer | Siemens®, Germany | General Electrics®, USA | Siemens®, Germany | SUVmax | 0.94 | 0.07 |
| FDG activity | 4.5 MBq/kg | 3.5 MBq/kg | 3.5 MBq/kg | SUVmean | 0.82 | 0.07 |
| Acquisition time after injection | Around 60 min | Around 60 min | Around 60 min | TMTV | 0.94 | 0.11 |
| PET voxel sizes | 4×4×2 mm | 2.7x2.7x3.3 mm | 4×4×2 mm | TLG | 0.94 | 0.21 |
| Image interpolation | No | No | No | TMTS | 0.87 | 0.13 |
| Time of flight | No | Yes | Yes | TVSR | 0.82 | 0.16 |
| Reconstruction | OSEM (4 iterations and 8 subsets) | OSEM ( 2 iterations and 24 subsets) | OSEM (3 iterations and 21 subsets) | TumBB | 0.82 | <0.001* |
| Gaussian post-filter | 5 mm | 6.4 mm | 5 mm | Dmax | 0.87 | <0.001* |
| Corrections for scatter and | Yes | Yes | Yes | nROI | 0.82 | 0.08 |
| Attenuation correction based on CT-derived μ-maps | Yes | Yes | Yes | itErosion | 0.87 | 0.02* |
| CT scan | 100 to 120 kV, with an intensity modulation system | 100 to 120 kV, with an intensity modulation system | 100 to 120 kV, with an intensity modulation system | medPCD | 0.82 | 0.02* |
| Point-spread function modeling | No | Yes | Yes | medEdgeD | 0.94 | 0.07 |
| SUV normalization | patient’s weight | patient’s weight | patient’s weight | Non included* | PET/CT n°3, n=3 | PET/CT n°3, n=2 |

*****Parameters from PET/CT n°3 (Biograph40) are not included in the Wilcoxon tests comparing the PET parameter’s distributions due to the low number of examiantions performed with this machine.
