# Supplementary material for: Correlations between baseline 18F-FDG PET tumour parameters and circulating DNA in diffuse large B cell lymphoma and Hodgkin lymphoma
Source: EJNMMI Res. 2020 Oct 7;10:120. doi: 10.1186/s13550-020-00717-y (PMC7541805; doi:10.1186/s13550-020-00717-y)

**Supplemental figure 1**: Plot representing distribution of four PET parameters (TumBB, Dmax, itErosion, medPCD) acquired on PET/CT n°1 and n°2 before and after harmonization by ComBat with Wilcoxon-Mann-Whitney test p-value.


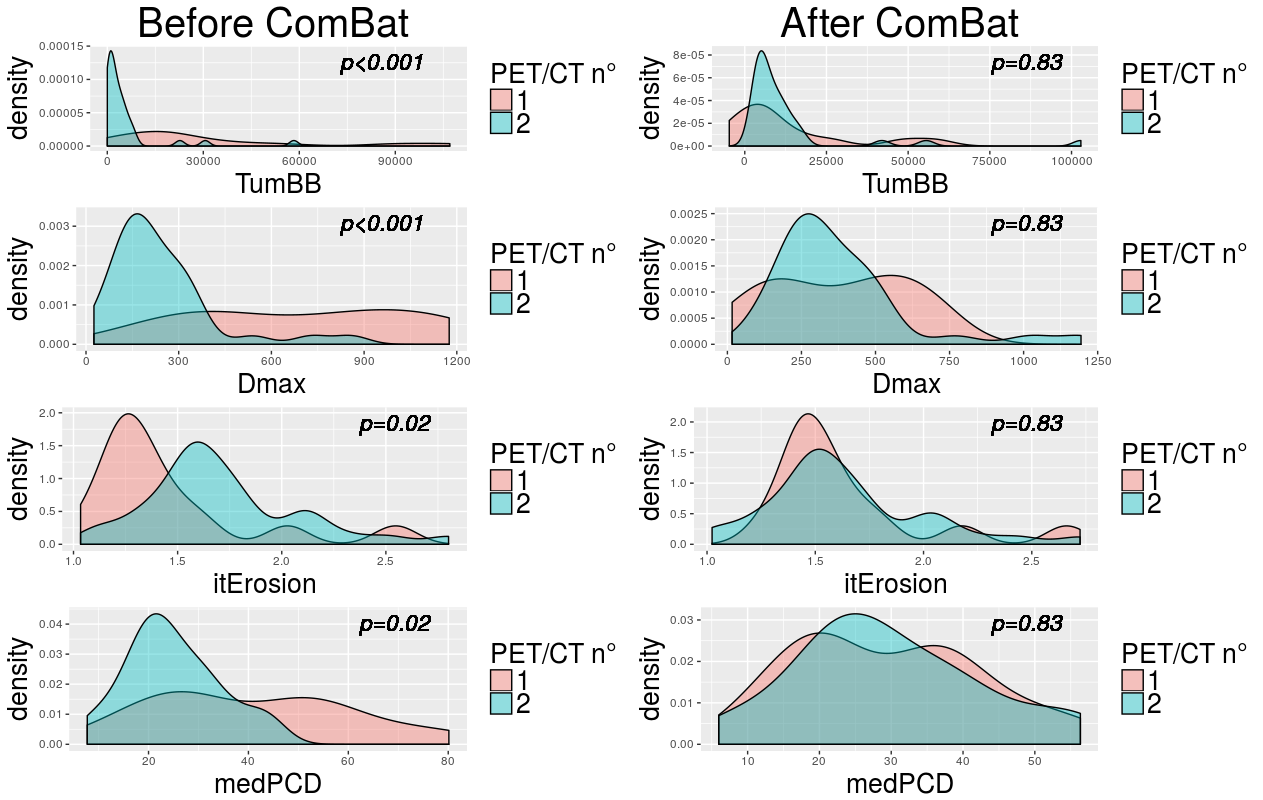

Supplement: Supplementary file 2 — Additional file 2: Figure 1. Plot representing distribution of four PET parameters (TumBB, Dmax, itErosion, medPCD) acquired on PET/CT n°1 and n°2 before and after harmonization by ComBat with Wilcoxon–Mann–Whitney test p-value. [file 13550_2020_717_MOESM2_ESM.doc]
