# Supplementary material for: Correlations between baseline 18F-FDG PET tumour parameters and circulating DNA in diffuse large B cell lymphoma and Hodgkin lymphoma
Source: EJNMMI Res. 2020 Oct 7;10:120. doi: 10.1186/s13550-020-00717-y (PMC7541805; doi:10.1186/s13550-020-00717-y)

**Supplemental figure 2**: Plot representing Spearman’s correlations (*p*<0.05 controlled by Benjamini-Hochberg correction, else blank) between PET parameters for DLBCL and cHL.


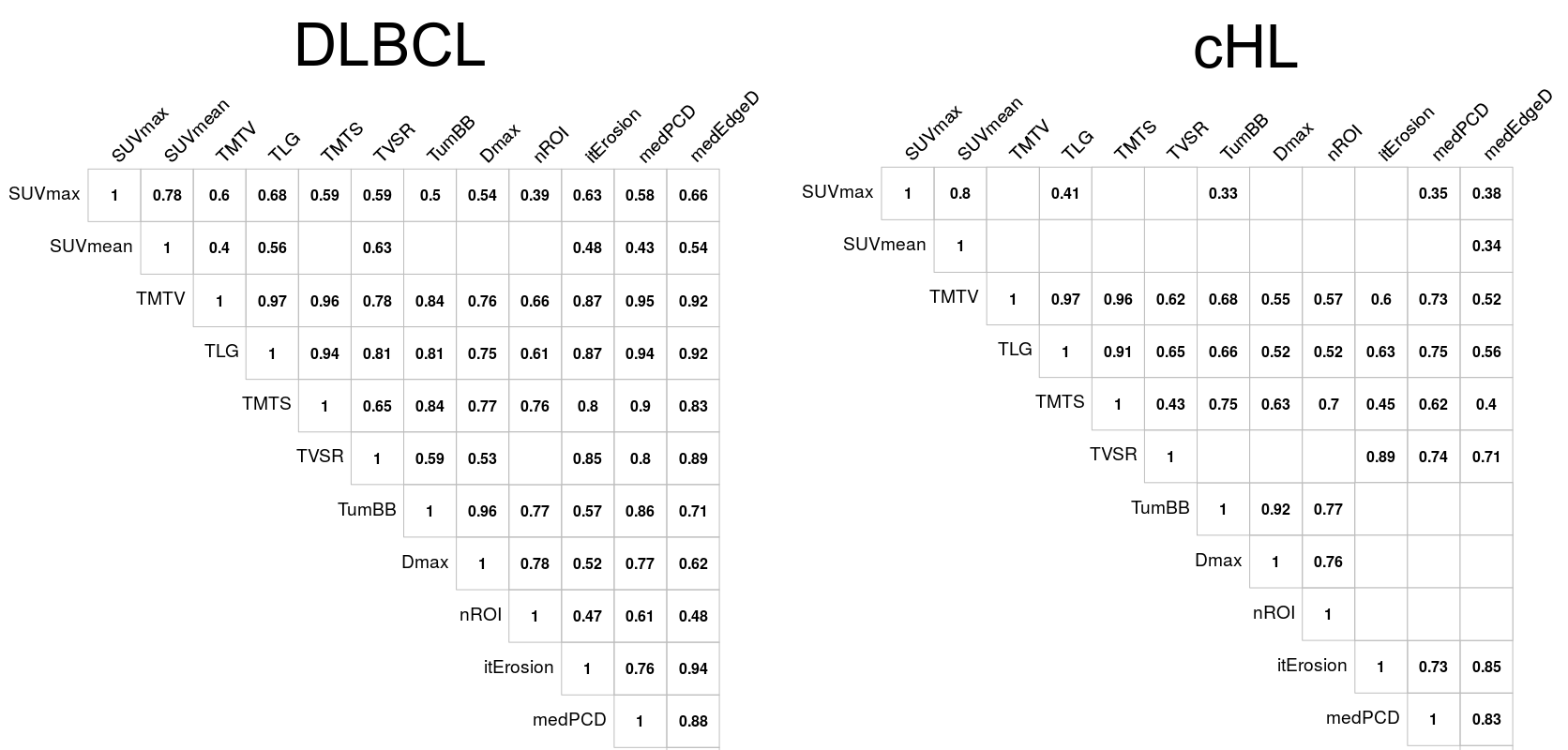

Supplement: Supplementary file 3 — Additional file 3: Figure 2. Plot representing Spearman’s correlations (p < 0.05 controlled by Benjamini–Hochberg correction, else blank) between PET parameters for DLBCL and cHL. [file 13550_2020_717_MOESM3_ESM.doc]
