# Supplementary material for: Correlations between baseline 18F-FDG PET tumour parameters and circulating DNA in diffuse large B cell lymphoma and Hodgkin lymphoma
Source: EJNMMI Res. 2020 Oct 7;10:120. doi: 10.1186/s13550-020-00717-y (PMC7541805; doi:10.1186/s13550-020-00717-y)
Supplement: Supplementary file 4 — Additional file 4: Table 2. Univariate linear regression between twelve PET parameters (SUVmax, SUVmean, TMTV, TLG, TMTS, TVSR, TumBB, Dmax, nROI, itErosion, medPCD, medEdgeD) and two blood DNA parameters ([cfDNA], [ctDNA]) for DLBCL and cHL with adjusted R-squared (R²). p values controlled by Benjamini–Hochberg correction. [file 13550_2020_717_MOESM4_ESM.doc]

**Supplemental table 2.** Univariate linear regression between twelve PET parameters (SUVmax, SUVmean, TMTV, TLG, TMTS, TVSR, TumBB, Dmax, nROI, itErosion, medPCD, medEdgeD) and two blood DNA parameters ([cfDNA], [ctDNA]) for DLBCL and cHL with adjusted R-squared (R ²). P-values controlled by Benjamini-Hochberg correction.

|  | [cfDNA] | | [ctDNA] | |
| --- | --- | --- | --- | --- |
|  | DLBCL | cHL | DLBCL | cHL |
| (1) SUVmax | 5.93e03+x*748 | 8.83e03+x*321 | -1.97e03+x*342 | 498-x*0.71 |
|  | [R²=0.009] (p=0.30) | [R²=-0.01] (p=0.72) | [R²=0.19] (p=0.21) | [R²=-0.03] (p=0.99) |
| (2) SUVmean | 1.39e04+x*845 | 1.32e04+x*102 | 2.77e03+x*274 | 801-x*54 |
|  | [R²=-0.03] (p=0.59) | [R²=-0.02] (p=0.95) | [R²=-0.03] (p=0.65) | [R²=-0.01] (p=0.99) |
| (3) TMTV | 5.56e03+x*22 | 8.26e03+x*20 | -2.94e02+x*7.1 | 2.03e02+x*0.90 |
|  | [R²=0.48] (p<0.001) | [R²=0.07] (p=0.09) | [R²=0.38] (p=0.006) | [R²=0.15] (p=0.044) |
| (4) TLG | 5.93e03+x*1.9 | 7.32e03+x*4.2 | -212 + x*6.2e-01 | 1.69e+02+x*0.19 |
|  | [R²=0.46] (p<0.001) | [R²=0.08] (p=0.08) | [R²=0.36] (p=0.006) | [R²=0.15] (p=0.044) |
| (5) TMTS | 6.73e03+x*17.5 | 6.05e03+x*14 | -329+x*6.0 | 2.02e+02+x*0.44 |
|  | [R²=0.37] (p=0.001) | [R²=0.15] (p=0.049) | [R²=0.34] (p=0.006) | [R²=0.14] (p=0.046) |
| (6) TVSR | 591+x*3.13e04 | 1.53e04-x*333 | -1.642e03+x*1.03e03 | 22+x*101 |
|  | [R²=0.11] (p=0.07) | [R²=-0.02] (p=0.95) | [R²=0.07] (p=0.17) | [R²=0.02] (p=0.33) |
| (7) TumBB | 1.2e04+x*7.01e-01 | 9.167e03+x*0.36 | 2.74e+03+x*0.19 | 2.77e02+x*1.39e-02 |
|  | [R²=0.17] (p=0.03) | [R²=0.11] (p=0.05) | [R²=0.07] (p=0.17) | [R²=0.16] (p=0.044) |
| (8) Dmax | 6.39e03+x*40 | 2.13e03+x*31 | 1.02e03+x*11 | -12.54+x*1.25 |
|  | [R²=0.14] (p=0.046) | [R²=0.12] (p=0.049) | [R²=0.06] (p=0.17) | [R²=0.17] (p=0.044) |
| (9) nROI | 1.61e04+x*720 | 9.80e03+x*331 | 3.39e03+x*226 | 289+x*14 |
|  | [R²=0.07] (p=0.13) | [R²=0.01] (p=0.40) | [R²=0.04] (p=0.21) | [R²=0.026] (p=0.32) |
| (10) itErosion | -2.69e.04+x*2.32e04 | 2.05e04-x*4.14e03 | -1.06e04+x*7.63e03 | 357+x*80 |
|  | [R²=0.39] (p=0.001) | [R²=-0.02] (p=0.78) | [R²=0.33] (p=0.006) | [R²=-0.03] (p=0.99) |
| (11) medPCD | -3.34e03+x*522 | 7.16e03+x*223 | -2.98e03+x*166 | -52+x*18 |
|  | [R²=0.27] (p=0.008) | [R²=0.002] (p=0.52) | [R²=0.20] (p=0.04) | [R²=0.07] (p=0.16) |
| (12) medEdgeD | -8.91e03+x*708 | 1.29e04+x*30 | -4.85e03+x* 233 | 481+x*0.2 |
|  | [R²=0.22] (p=0.02) | [R²=-0.02] (p=0.94) | [R²=0.19] (p=0.04) | [R²=-0.03] (p=0.99) |
